# Soil Fungal:Bacterial Ratios Are Linked to Altered Carbon Cycling

**DOI:** 10.3389/fmicb.2016.01247

**Published:** 2016-08-09

**Authors:** Ashish A. Malik, Somak Chowdhury, Veronika Schlager, Anna Oliver, Jeremy Puissant, Perla G. M. Vazquez, Nico Jehmlich, Martin von Bergen, Robert I. Griffiths, Gerd Gleixner

**Affiliations:** ^1^Department of Biogeochemical Processes, Max Planck Institute for BiogeochemistryJena, Germany; ^2^Centre for Ecology and HydrologyWallingford, UK; ^3^Department of Proteomics, Helmholtz Centre for Environmental ResearchLeipzig, Germany; ^4^Department of Metabolomics, Helmholtz Centre for Environmental ResearchLeipzig, Germany; ^5^Institute of Biochemistry, Faculty of Biosciences, Pharmacy and Psychology, University of LeipzigLeipzig, Germany; ^6^Department of Life Sciences and Chemistry, Aalborg UniversityAalborg, Denmark

**Keywords:** bacteria, fungi, litter decomposition, proteomics, RNA sequencing, soil carbon, stable isotopes

## Abstract

Despite several lines of observational evidence, there is a lack of consensus on whether higher fungal:bacterial (F:B) ratios directly cause higher soil carbon (C) storage. We employed RNA sequencing, protein profiling and isotope tracer techniques to evaluate whether differing F:B ratios are associated with differences in C storage. A mesocosm ^13^C labeled foliar litter decomposition experiment was performed in two soils that were similar in their physico-chemical properties but differed in microbial community structure, specifically their F:B ratio (determined by PLFA analyses, RNA sequencing and protein profiling; all three corroborating each other). Following litter addition, we observed a consistent increase in abundance of fungal phyla; and greater increases in the fungal dominated soil; implicating the role of fungi in litter decomposition. Litter derived ^13^C in respired CO_2_ was consistently lower, and residual ^13^C in bulk SOM was higher in high F:B soil demonstrating greater C storage potential in the F:B dominated soil. We conclude that in this soil system, the increased abundance of fungi in both soils and the altered C cycling patterns in the F:B dominated soils highlight the significant role of fungi in litter decomposition and indicate that F:B ratios are linked to higher C storage potential.

## Introduction

The soil organic carbon (SOC) pool is larger than global vegetation and the atmospheric pool combined, and the exchanges of C between the soil and the atmosphere are quantitatively relevant for the terrestrial C cycle (Amundson, 2001; Lal, 2010). Soil microorganisms due to their high biomass and activity largely control soil C cycling thus acting as gatekeepers for soil-atmosphere C exchange (Schimel and Schaeffer, 2012; Gleixner, 2013). This regulation is a result of a balance between the rate of microbial decomposition and stabilization of organic C in soil. This balance can shift under altered environmental conditions (Davidson and Janssens, 2006) and hence, predicting consequences of the microbial physiological regulation on soil C processes is critical in projecting future global warming and mitigating atmospheric CO_2_ levels (Billings and Ballantyne, 2013; Schimel, 2013).

Earth system models projecting soil C stock changes often do not explicitly consider key biogeochemical mechanisms like microbial physiological responses (Allison and Martiny, 2008; Zhou et al., 2011; Wieder et al., 2013). The reason for the exclusion of microbial physiology in climate-carbon models is largely the lack of mechanistic understanding of the feedback responses of complex soil microbial communities (Bardgett et al., 2008). One way of reducing this complexity and yet include some details regarding microbial processes into models is to incorporate ecologically meaningful and functionally relevant microbial indicators. A widely considered proxy microbial indicator is based on the sub-division of microbes into major decomposer groups, namely the fungi and bacteria, indexed as fungal:bacterial (F:B) ratio. The ratio has been extensively used in soil ecology particularly in the context of land management and its effects on soil carbon sequestration (Bardgett et al., 1996; Strickland and Rousk, 2010).

Intensively managed farmed soils often exhibit lower F:B biomass ratios when compared with more extensively managed soils; a phenomena thought to be due to tillage, high rates of fertilization and decreasing C:N ratio favoring bacteria (Bardgett et al., 1996; Bailey et al., 2002; Sinsabaugh et al., 2013). Lower fungal biomass has been linked to lower capacity of such soils to sequester C. A shift toward a fungal dominance in the microbial community is thought to enhance organic C accumulation and decrease its turnover rate due to enhanced fungal mediated soil aggregation and/or changes in the physiology of the microbial biomass (Six et al., 2006). For instance, fungi are thought to express a broader suite of enzymes capable of transforming and stabilizing inputs; and fungal biomass has greater C:N ratio which results in increased carbon use efficiency (Strickland and Rousk, 2010; Waring et al., 2013). Despite sufficiently established associative linkages between environmental change factors like land use, F:B ratios and carbon storage; there is still a lack of experimental studies demonstrating the underlying physiological and genetic mechanisms underpinning these field observations. Moreover, there is no direct evidence to implicate fungi in enhancing soil C storage, due to difficulties in manipulating complex soil communities and technical challenges in measuring *in situ* activities. RNA and protein profiling gives a better picture of the active microbial community compared to PLFA or DNA based taxonomic assays (Strickland and Rousk, 2010). Sequencing of total extracted RNA without amplifying specific rRNA regions gives a quantitative assessment of the taxonomic diversity of total microbial communities (Urich et al., 2008). This allows indexing of F:B ratios that is otherwise not possible with amplicon sequencing based taxonomic assays. Similarly, profiling of total extracted proteins allows quantification of relative changes in active fungal and bacterial communities. To date there have been no attempts to use these new technologies to advance current understanding of the role of fungal dominance in enhancing soil C storage.

In this study, we describe a mesocosm experiment that aimed to explicitly link microbial community differences, specifically in soil F:B ratio, to the soil’s potential to store C. We performed a litter addition experiment on two soils that differed only in their microbial community composition but not in physical and chemical properties. Previous work has shown that differences in the F:B ratio of these soils were due to small field-manipulated differences in the aboveground plant diversity; PLFA-derived bacterial biomass was largely similar in the two soils while clear differences were discernable in the fungal biomass. Following the addition of ^13^C labeled plant litter to these soils, RNA sequencing and protein profiling were applied to assess changes in the taxonomic and functional characteristics of the microbial community. The tracer C was followed into microbial biomass, respired CO_2_ and soil organic matter to complete mass balance of the applied substrate and assess concomitant changes in C storage.

## Materials and Methods

### Soil System

To investigate the link between microbial community structures and soil C cycling we chose two soils from the Jena Biodiversity Experiment, a large-scale grassland diversity experiment established in 2002 in Jena, Germany (Roscher et al., 2004). The soils were chosen based on PLFA-derived microbial community composition from 2007 (Lange et al., 2014), such that the soils were similar in their physical and chemical properties but differed in their microbial community structure, and specifically their F:B ratio. Therefore, the two soils were named “low F:B soil” and “high F:B soil.” This nomenclature has been followed throughout this report. Both soils had similar soil texture (sand 44.6%, silt 39.6%, clay 15.8%) and pH (7.75). Soil C, N and C:N ratio were also very similar at 2, 0.2, and 10.2%, respectively (**Supplementary Figure S1**). The two soils differed in the number of plant species (four in Low F:B soil, eight in high F:B soil) but not in the number of plant functional groups (one grass, one small herb, one tall herb, and one legume in both soils). Soil was collected from 0 to 10 cm depth (from three spatially replicated plots for each soil class) using stainless steel cores in April 2014, sieved (<2 mm), all visible roots removed, homogenized and stored at 4°C for no longer than 12 days until the mesocosms were established.

### Plant Litter Production

^13^C labeled plant foliar litter was produced in glass chambers in the greenhouse by growing *Dysphania ambrosioides* (formerly *Chenopodium ambrosioides*), a temperate herb with vermiculite as a substrate. A constant supply of ^13^CO_2_ (∼1 atom% ^13^C) at 400 ppm was maintained in continuous flow-through growth chambers. The mesocosms were uniformly watered with an automated irrigation system; and additional light was provided 12 h per day. After 3 months of growth, plants were harvested; leaves were separated, dried at 40°C, shredded and ground in a ball-mill. The product was subsequently sieved (<500 μm) and the finer powder was used for soil application. An aliquot was taken for ^13^C analysis on elemental analyzer coupled to an isotope ratio mass spectrometer (EA model CE 1100 coupled on-line via a Con Flo III[27] interface with a Delta + isotope ratio mass spectrometer; all supplied by Thermo Fisher Scientific, Germany). Fifty milligram of plant leaf litter (2.1 ± 0.1 atom%) was added to each soil mesocosm that was equivalent to 531.3 ± 21.5 μg^13^C.

### Experimental Setup

Pots (200 ml) were filled with 50 g of sieved and homogenized soil and left in the greenhouse for 3 weeks for acclimatization. Fifty milligram of powdered ^13^C enriched foliar litter was then added to each soil mesocosm and gently mixed. To not create a major disturbance in the soil system that could lead to big changes in microbial physiology, the C amendment was kept small (2.5% organic C added relative to soil C). Litter was shredded and powdered to exclude the effect of particle size, thus giving a level playing field to fungi and bacteria, the major soil decomposers. Uniform soil moisture content was maintained by spraying sterilized water throughout the entire experimental period. Three mesocosms per soil class (low F:B and high F:B) were destructively harvested in a time series at 6 h, 1, 3, 7, 14, 21, and 30 days after litter addition. An aliquot of soil was stored at -80°C prior to nucleic acid and protein extraction. In addition, soil for microbial biomass chloroform fumigation extraction and respiration measurements was stored at -20°C. A smaller aliquot for ^13^C measurement of bulk soil organic matter was dried at 40°C.

### PLFA Analysis

PLFA-derived microbial community composition was reassessed with soil from the same field plots (*n* = 3) sampled in April 2012. Microbial lipids were extracted from approximately 50 g (dry weight) of soil according to a modified Bligh and Dyer extraction protocol (Bligh and Dyer, 1959; Kramer and Gleixner, 2008). Extractions were carried out using a mixture of chloroform, methanol and 0.05 M phosphate buffer (pH 7.4) (1:2:0.8 v:v:v). Extracted lipids were separated into neutral lipids, phospholipids and glycolipids using silica columns. Fatty acid methyl esters (FAMEs) were then isolated by mild alkaline hydrolysis and methylation of fatty acids, followed by the removal of unsubstituted FAMEs. PLFAs were then separated into saturated fatty acids (SATFA), monounsaturated fatty acids (MUFA) and polyunsaturated fatty acids (PUFA). All extracts were dried under a nitrogen stream, resuspended in a 200 μL stock solution containing n19:0 in isooctane as internal standard. Gas chromatography flame ionization detector (GC-FID, Hewlett Packard HP 6890 series GC-System coupled with a FID; Agilent Technologies, Palo Alto, CA, USA) was used to quantify the different PLFAs. The following identified PLFAs were used as bacterial markers: 14:0, 15:0, 16:0, 17:0, 18:0, 14:0i, 15:0i, 15:0a, 16:0i, 17:0i, 17:0a, 16:0(10Me), 17:0(10Me), 18:0(10Me), 17:0cy, 19:0cy, 15:1, 16:1ω7, 16:1ω5, 16:1, 17:1, 18:1ω7 and 18:1ω9; while 18:2ω6,9 was used as a fungal marker.

### RNA Sequencing

Microbial RNA was extracted from 2 g soil using PowerSoil total RNA isolation kit according to manufacturer’s instructions (MO BIO Laboratories, UK). Soils from before and a week after litter application (*n* = 3) were processed for RNA sequencing; 1-week time point was chosen based on microbial respiratory fluxes that were highest at this time point. Following elution in nuclease-free water, the product was treated with RQ1 RNase-free DNase (Promega, UK) to remove co-extracted DNA followed by RNA clean up using RNeasy MiniElute spin columns (Qiagen, UK). The purity and concentration of RNA was assessed using R6K ScreenTape on Agilent TapeStation (Agilent Technologies, UK). RNA was then prepared for sequencing with Illumina MiSeq (Illumina, UK). We sequenced the total RNA (rRNA, mRNA and other RNA species) in order to get both taxonomic and functional information (Urich et al., 2008). Sequencing library was prepared using NEBNext mRNA Library Prep Master Mix Set for Illumina and NEBNext Multiplex Oligos for Illumina according to manufacturer’s instructions (New England BioLabs, UK). Briefly, 18 μl RNA was fragmented, fragments were purified using RNeasy MiniElute spin columns, followed by reverse transcription using random primers and purification of the resulting double stranded cDNA with 1.8X Agencourt AMPure XP beads (Beckman Coulter, UK). The next steps included end repair, dA-tailing and adaptor ligation of the cDNA library (each step was followed by purification with AMPure beads). The adaptor-ligated DNA was then size selected for 250 bp using E-Gel (Invitrogen, UK) and PCR enriched (10 PCR cycles) using universal PCR primer and unique index primers for each sample. The PCR product was purified with AMPure beads, 1% agarose gel (to remove primer dimers) and QIAquick gel extraction kit. The quality of the purified PCR product was assessed using D1000 ScreenTape on Agilent TapeStation and its concentration was estimated using qPCR. A multiplexed cDNA library was run on Illumina MiSeq. Forward and reverse sequence reads were joined, quality filtered and annotated with the Metagenomics Rapid Annotation using Subsystems Technology (MG-RAST) server version 3.6. Taxonomic and functional classification was performed using the M5NR and SEED database, respectively, with a maximum *e*-value cut-off of 1e-5; minimum identity cut-off of 60% and minimum length of sequence alignment of 15 nucleotides. Raw sequences were deposited in MG-RAST under the following accession numbers: 4601517.3, 4601521.3, 4601522.3 (Low F:B soil before litter addition), 4601523.3, 4601524.3, 4601525.3 (Low F:B soil 7 days after litter addition), 4601526.3, 4601527.3, 4601528.3 (High F:B soil before litter addition), 4601518.3, 4601519.3, 4601520.3 (High F:B soil 7 days after litter addition).

### Metaproteomic Analysis

For proteomic analysis of microbial communities, triplicate samples from each time point were pooled together and a composite sample for each F:B ratio class from time 0, 1, 7, and 30 days was used for protein extraction. Five g soil was homogenized with 5 ml SDS extraction buffer (1.25% w/v SDS, 0.1 M Tris-HCl, pH 6.8, 20 mM DTT) by shaking for 1 h. Glass and zirconium beads were added to the homogenized soil before ultra-sonication (two cycles for 3 min, 1 min on ice) centrifuged at 13000 *g* for 10 min at 4°C. Three ml buffered phenol (pH 8) was added to the filtrate and subjected to shaking for 15 min followed by centrifugation (13000 *g*, 4°C) for 10 min. The aqueous supernatant was collected, shaken with 3 mL buffered phenol (pH 8) and centrifuged (13000 *g*, 4°C) for 30 min. The aqueous phase was washed again with 3 ml buffered phenol (pH 8) and shaken for 15 min followed by centrifugation (13000 *g* at 4°C) for 10 min. The phenol phases from both rounds were combined and washed twice with 3 mL Milli-Q water for 15 min. Another centrifugation (13000 *g*, 4°C, 30 min) was performed to recover the lower phenol phase with proteins, which were then precipitated with fivefold volume of 0.1 M ammonium acetate dissolved in methanol at -20°C overnight. The precipitated proteins were recovered by centrifugation (13000 *g*, 4°C, 30 min), the supernatant was discarded and the pellet was washed once with cold methanol and twice with cold acetone. After centrifugation (13000 *g*, 4°C, 30 min), the pellet was air-dried and stored at -20°. The protein pellet was resuspended in 5 μl Milli-Q water and subjected to 1D SDS-PAGE in a 12% polyacrylamide gel to remove interfering co-extracted substances. Lane slices were cut into pieces of approximately 1 mm^2^ and subjected to immediate tryptic digestion with sequencing grade-modified trypsin (Promega, Germany).

Proteolytically cleaved peptides were separated prior to mass spectrometric analyses by reverse phase nano HPLC on a nano-HPLC system (nanoACQUITY, Waters, Milford, MA, USA) coupled online for analysis with an LTQ Orbitrap Velos mass spectrometer (Thermo Fisher Scientific, San Jose, CA, USA) equipped with a nano electrospray ion source operated with PicoTip Emitters (New Objective, Woburn, MA, USA). Peptide separation was performed using a 180 min gradient from 2 to 40% acetonitrile, 0.1% formic acid, followed by a 15 min gradient from 40 to 85% acetonitrile, 0.1% formic acid on a C18 column (nanoAcquity UPLC column, C18, 75 μm × 150 mm, 1.7 μm, Waters) with a flow rate of 300 nl/min and a column temperature of 40°C. Continuous scanning of eluted peptide ions was carried out between 375 and 1,500 m/z, automatically switching to MS/MS collision induced dissociation (CID) mode. Raw data from the MS instrument were processed using Proteome Discoverer v1.4.1.14 (Thermo Fisher Scientific). MS data were searched against a FASTA-formatted database (protein-coding sequences of bacteria, fungi and archaea) using the Mascot algorithm. Database searches were performed with carbamidomethylation on cysteine as fixed modification and oxidation on methionine as variable modification. Enzyme specificity was selected to trypsin with up to two missed cleavages allowed using 10 ppm peptide ion and 0.6 Da MS/MS tolerances. Only peptides with a false discovery rate (FDR) <1% estimated by fixed value peptide spectral match validation, and only rank 1 peptides were accepted as identified. To further assess the function of the different community members, we assigned the identified proteins to clusters of orthologous groups (COG). The lowest common phylogenetic ancestor of each protein group was classified using the PROPHANE pipeline^1^

### ^13^C Analysis of Respired CO_2_, Microbial Biomass and Soil Organic Matter

For respiration measurements, a smaller soil aliquot (1 g) was placed in a 10 mL glass vial and incubated overnight (for ∼16 h) in the dark at room temperature (21°C). Respired ^13^CO_2_ collected in the headspace was measured using a gas chromatography isotope ratio mass spectrometer (GC-IRMS, Delta^+^ XL, Thermo Fisher Scientific, Germany) coupled to a PAL-autosampler (CTC Analytics) with general purpose (GP) interface (Thermo Fisher Scientific, Germany). In addition to analyzing the destructively sampled soils, ^13^C content in respired CO_2_ was also measured more intensively at regular frequencies of about an hour in the first 24 h to monitor the initial dynamics. This was achieved by repeated withdrawing of 10 μL of headspace air from glass vials with 1 g of soil with litter amendment. Soil microbial biomass was obtained using a slightly modified version of the chloroform fumigation extraction (CFE) method (Vance et al., 1987; Malik et al., 2013). Soil (7 g) was fumigated with chloroform gas and organic carbon was extracted using 0.05 M K_2_SO_4_ solution. A non-fumigated control was also maintained. Extracts were analyzed in the bulk (μEA) mode on an HPLC-IRMS (HPLC system coupled to a Delta^+^ XP IRMS through an LC IsoLink interface; Thermo Fisher Scientific, Germany). More details are given elsewhere (Malik et al., 2013). Total microbial biomass content was estimated by using the correction factor (0.45) that accounts for the extraction inefficiency. For ^13^C analysis of bulk soil organic matter, dried soil was ground using a ball-mill and carbon isotope measurement was performed on an elemental analyzer coupled to an isotope ratio mass spectrometer.

The net ^13^C enrichment in different pools was estimated as Δδ^13^C value which signifies the change in δ^13^C value after incubation with ^13^C labeled litter relative to the unlabeled pre-incubation sample. We then calculated the absolute amounts of ^13^C in different pools which is expressed in ng ^13^C g^-1^ of soil (Boschker et al., 1998). To monitor the incorporation of litter derived ^13^C in soil, ^13^C amounts in microbial biomass were subtracted from those in SOM to get the ^13^C excess in non-living SOM. This index denotes microbial necromass and residues incorporated into SOM but could also simply be undecomposed labeled litter. A mass balance of the total applied litter-derived ^13^C (531.3 ± 21.5 μg^13^C per soil mesocosm) into the different soil pools was performed at 30 days after litter addition. Total respiratory loss of litter-derived carbon was measured by integrating the area under the curve of ^13^C excess in respired CO_2_ across the time series. Total ^13^C excess in soil microbial biomass and non-living SOM at the end of the experimental period was calculated using the respective ng ^13^C g^-1^ soil values at 30 days after litter addition.

### Statistical Analysis

PLFA, RNA and protein based F:B ratios were calculated using lipid quantities, proportion of sequences and protein abundances, respectively. Percent proportion of fungi relative to bacteria was expressed as below:

$$\%proportion\,\;F:B=\frac{Fungal\,\;index}{Bacterial\,\;index}\times 100$$

We used percent proportion of fungi relative to bacteria in the text as it provides a better picture of the differences with lesser decimal points than the absolute F:B ratio.

Variance in F:B ratio derived from PLFAs was statistically tested using single factor ANOVA (*n* = 3). Statistical significance in variance of RNA Sequencing-derived community ratios across community types and litter treatment was ascertained using Fischer’s least significant difference test (*n* = 3); preformed using XLSTAT version 2015.4. To identify patterns in microbial community composition, a principal component analysis (PCA) based on Hellinger-transformed RNA-Seq taxonomic data was performed (Legendre and Gallagher, 2001). We used a permutational multivariate ANOVA (ADONIS) based on Euclidean distance to test the effect of litter addition, F:B ratio variability and the interaction of both on microbial community structure (*n* = 3). RNA-Seq data were normalized by total number of sequences to obtain relative abundances. Fold change in relative abundance of major bacterial and fungal phyla on litter addition was calculated by dividing the relative abundance of each phylum after litter input by that before addition. Statistical significance of this fold change in RNA-Seq derived relative abundance was calculated using two sided *t*-test (equal variance) on STAMP software version 2.1.3 (Parks et al., 2014). The global effect of litter addition and F:B ratio and their interactions on major taxa at domain and phylum level were assessed by repeated measures ANOVA. To test the statistical significance in the variability of proteomics-based F:B ratio, we averaged the F:B ratio obtained at different time points (*n* = 4) and tested for variance using single factor ANOVA.

Kruskal–Wallis tests (non-parametric, *n* = 3) was used to examine significant differences in litter-derived ^13^C excess in microbial biomass, respired CO_2_ and non-living SOC between the two soils at each sampling time point. The global effect of sampling time and soil class; and their interactions on the measured parameters were assessed by repeated measures ANOVA. Sampling time and soil class were the fixed factors, while soil replicate was added as a random factor. Assumptions of normality and homoscedasticity of the residuals were verified visually using diagnostic plots and a Shapiro–Wilk test. These statistical analyses were performed under the R environment software 3.2.5 (R Development Core Team, 2013), using the R package NLME (Pinheiro et al., 2014).

## Results

### PLFA Based F:B Ratio

**FIGURE 1:**
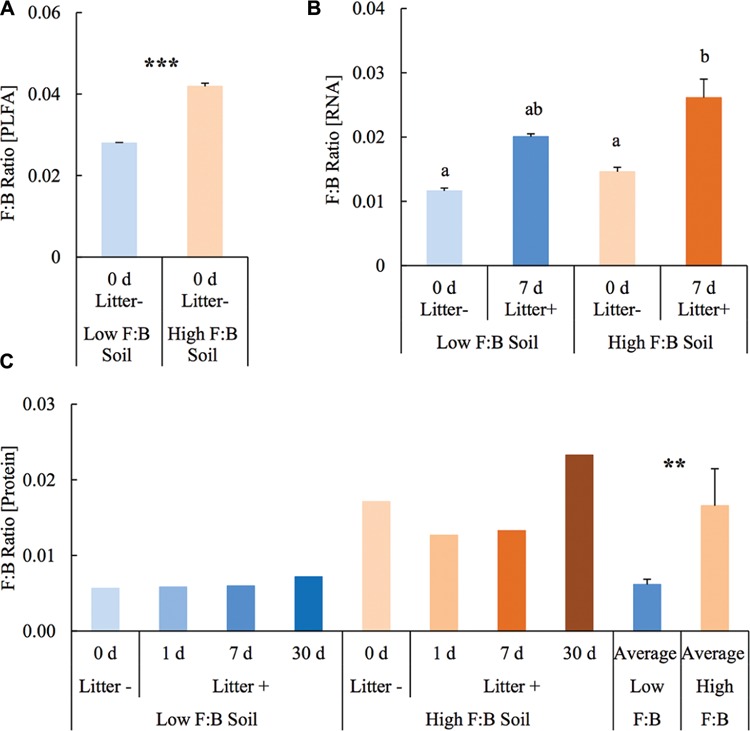
**Fungal:bacterial ratio estimated using different techniques: (A)** PLFA profiling: Error bars represent standard error (*n* = 3) and asterisks represent statistically significant variance (ANOVA *p*-values; ^∗^*p* < 0.05, ^∗∗^*p* < 0.01, ^∗∗∗^*p* < 0.001). PLFA analysis was done on field soils before setting up the mesocosms. **(B)** RNA-Seq: error bars represent standard error (*n* = 3), lowercase letters from Fischer LSD test; treatments sharing a letter are not significant. **(C)** Proteomics: values were derived from composite samples of replicates at each time point and the average value was derived from all time points for low and high F:B soils. Asterisks represent statistically significant variance (ANOVA *p*-values; ^∗^*p* < 0.05, ^∗∗^*p* < 0.01, ^∗∗∗^*p* < 0.001).

Before setting up the mesocosm experiment, the microbial community composition from PLFA biomarkers was reassessed with soils from the same diversity plots. Total bacterial PLFA content was 18.46 ± 0.05 and 23.55 ± 0.23 μg/g; while the fungal PLFA content was measured at 0.52 ± 0.002 and 0.99 ± 0.01 μg/g in low F:B and high F:B soil, respectively. The percent proportion of fungi relative to bacteria was estimated at 2.8 ± 0.01 and 4.2 ± 0.07 in low F:B and high F:B soil, respectively (**Figure 1A**). The PLFA-derived F:B ratio of high F:B soil microbial community was 1.5 times higher than that of low F:B soil (single factor ANOVA; *p* < 0.001).

### Soil Biodiversity from RNA Sequencing

**FIGURE 2:**
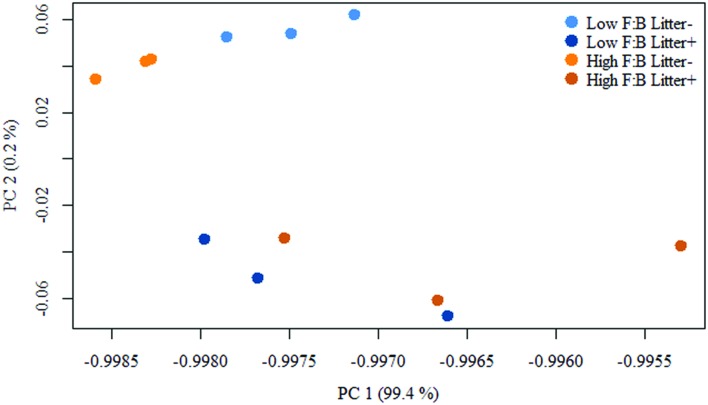
**Principal component analysis (PCA) of RNA-Seq derived microbial community composition.** Permutational multivariate ANOVA (ADONIS) was used to test treatment effect; F:B ratio of microbial communities: *p* = 0.09, litter input: *p* = 0.0002 and interaction of both: *p* = 0.16.

**FIGURE 3:**
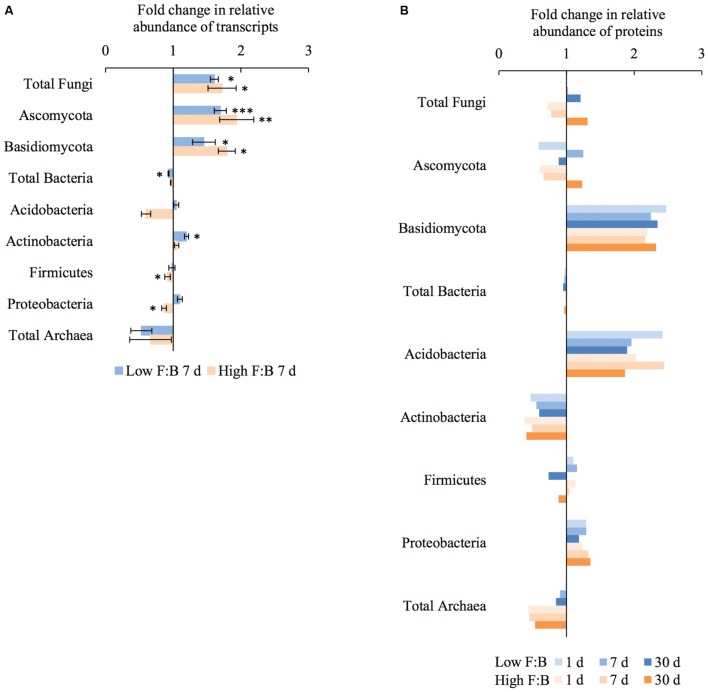
**Shifts in abundance of different microbial groups after litter addition at different time points obtained using: (A)** RNA-Seq: Error bars represent standard error (*n* = 3) and asterisks represent statistically significant fold change in relative abundance on litter addition (two sided *t*-test derived *p*-values; ^∗^*p* < 0.05, ^∗∗^*p* < 0.01, ^∗∗∗^*p* < 0.001); **(B)** Proteomics, values were derived from composite samples of replicates at each time point.

Total RNA sequencing (RNA-Seq) resulted in more than 27 million sequence reads. Taxonomic information from RNA-Seq was employed to estimate F:B ratio in the two soils before and a week after litter application. This PCR-free sequencing of total RNA (rRNA, mRNA and other RNA species) allowed us to quantitatively assess the taxonomic shifts in microbial communities in response to plant litter input. Bacterial RNA was far more dominant than fungal RNA, with the most abundant phyla detected by annotation of rRNA as well as functional genes being *Actinobacteria, Proteobacteria, Firmicutes, Bacteroidetes, Ascomycota, Planctomyscetes*, and *Cyanobacteria* in that order (**Supplementary Figures S2** and **S3**). PCA segregated the low and high F:B ratio microbial communities (ADONIS *p* = 0.09) but the clustering based on litter treatment was more pronounced and statistically significant (ADONIS *p* = 0.0002; **Figure 2**). The percent proportion of fungi relative to bacteria before litter addition was 1.2 ± 0.04 and 2 ± 0.04; and a week after litter addition it was 1.5 ± 0.07 and 2.6 ± 0.29 in low F:B and high F:B soil, respectively (**Figure 1B**). This shift toward increased fungi on litter addition in both soils was primarily due to increased abundance of all fungal phyla; and the fold change in relative abundance was bigger in high F:B soil (**Figure 3A**; **Supplementary Figure S4**). While the global effect of F:B ratio and litter input was smaller on bacterial relative abundance (ANOVA *p* = 0.52 and 0.02, respectively; **Supplementary Figure S3**), it was significant on the relative abundance of major fungal phyla: *Ascomycota* (ANOVA *p* = 0.005 and 0.0002, respectively) and *Basidiomycota* (ANOVA *p* = 0.016 and 0.07, respectively; **Supplementary Figure S4**). While total relative bacterial abundance did not change notably in both soils, certain groups did; most remarkably *Actinobacteria.* Actinobacterial relative abundance shifted on litter addition from 32.9 ± 0.2 to 39.4 ± 0.9% in low F:B soil and from 31.8 ± 0.7 to 33.3 ± 0.6 % in high F:B soil (**Supplementary Figure S2**). We estimated the *Actinobacteria*: rest of bacteria (A:RB) ratio, which shifted significantly on litter addition from 0.55 ± 0.01 to 0.84 ± 0.03 in low F:B soil and from 0.53 ± 0.02 to 0.61 ± 0.02 in high F:B soil (**Supplementary Figure S5**). Acidobacterial abundance halved on litter addition in high F:B soil; but no significant change was observed in low F:B soil. We also observed an increased abundance of plant RNA on litter addition. Its abundance increased from around 0.015% in both soils to 0.065 and 0.041% in low and high F:B soil, respectively.

### Proteomics-Based Taxonomic Assignment

Protein profiling also gave information on taxonomic shifts in microbial communities of the two soils before and 1, 7, and 30 days after litter application. Consistent with the transcriptomic data, bacterial proteins were by far the most dominant compared to fungi; and the most abundant bacterial phyla were *Proteobacteria* (22–31%), *Actinobacteria* (10–26%), *Firmicutes* (1.5–2.3%), and *Acidobacteria* (0.9–2.2%). The proteomics-derived percent proportion of fungi relative to bacteria in low F:B soil was 0.56, 0.58, 0.6 and 0.72 at 0, 1, 7, and 30 days after litter addition, respectively (**Figure 1C**). The percent proportion of fungi relative to bacteria in high F:B soil microbial community was 1.71, 1.27, 1.33, and 2.32 at 0, 1, 7, and 30 days after litter addition, respectively. Proteomics-derived F:B ratio averaged for all time points was significantly higher in high F:B soil (ANOVA; *p* = 0.005). The total bacterial abundance did not shift with litter addition, which is in line with the RNA-Seq results (**Figure 3B**). However, major shifts were observed in abundance of *Acidobacteria* and *Actinobacteria*; although the trends were not congruent with the RNA-Seq results. Protein profiling suggests that Acidobacterial abundance nearly doubled, whereas Actinobacterial abundance more than halved on litter addition. Increased total fungal abundance on litter addition was only observed 30 days after litter addition that was largely a reflection of shifts in abundance of *Ascomycota*, the most dominant fungal phyla. Interestingly, the abundance of *Basidiomycota* increased 2.2–2.4 fold on litter addition across the sampled time points. The abundance of *Archaeabacteria* decreased on litter addition in line with the RNA-Seq results.

### Tracer Distribution in Different Pools

**FIGURE 4:**
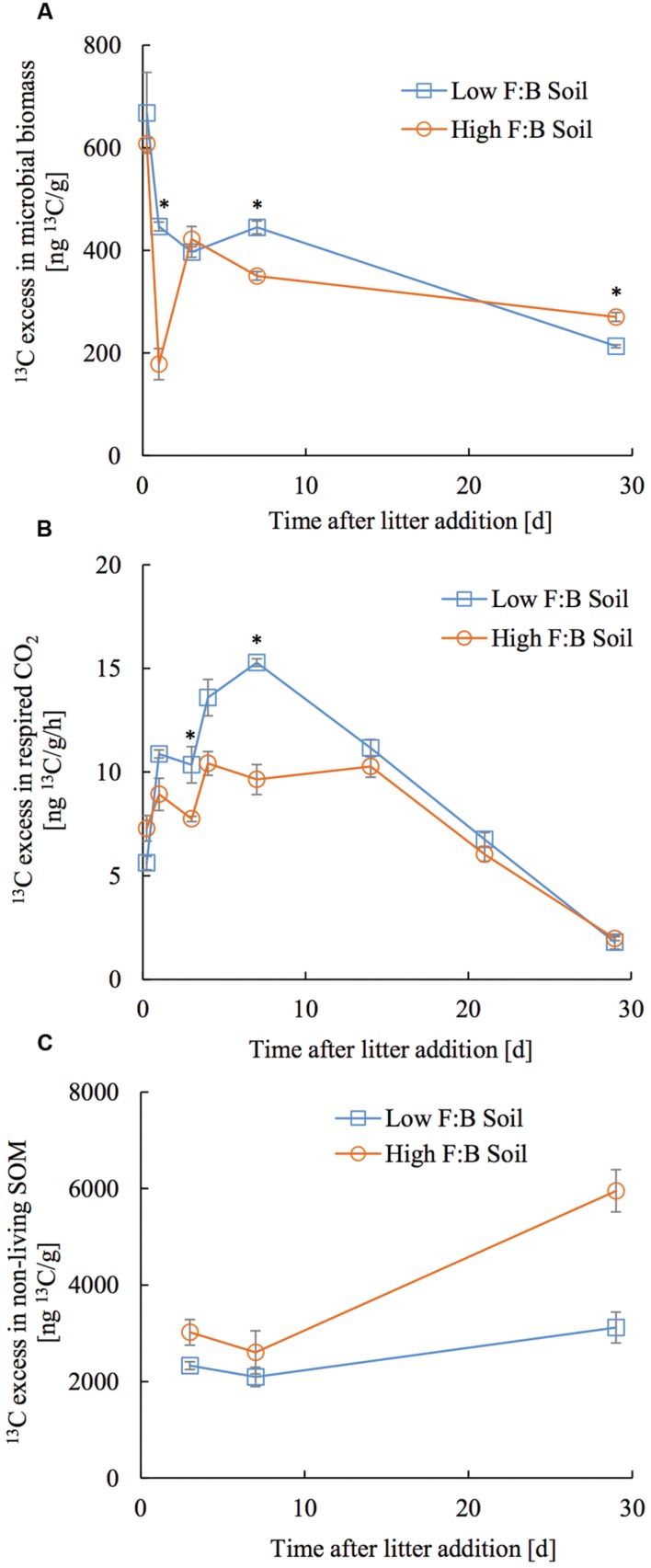
**Temporal trend in ^13^C excess in **(A)** microbial biomass, **(B)** respired CO_2_ and **(C)** non-living SOM (bulk SOM minus microbial biomass).** Error bars represent standard error (*n* = 3) and asterisks represent statistically significant fold change in relative abundance on litter addition (Kruskal–Wallis tests derived *p*-values; ^∗^*p* < 0.05, ^∗∗^*p* < 0.01 and ^∗∗∗^*p* < 0.001).

The applied tracer in the form of ^13^C labeled foliar litter was followed into the microbial biomass, its respired CO_2_ and bulk soil organic matter. ^13^C in microbial biomass obtained through CFE was 668 ± 78.7 and 607.6 ± 31.4 ng^13^C/g at 6 h after litter addition, which decreased to 213 ± 2.9 and 270 ± 8.6 ng^13^C/g at 30 days after litter addition in low and high F:B soil, respectively (**Figure 4A**). There was no consistent temporal trend in incorporation of ^13^C in microbial biomass of the two soils. The amount of microbially respired CO_2_ and its ^13^C excess were consistently lower in high F:B soil (**Figure 4B**; **Supplementary Figures S6** and **S7**). Basal respiration in low and high F:B soil was 1.1 ± 0.02 and 1 ± 0.03 μgC/g/h, respectively. It steadily increased on litter addition to reach maxima in 7 days (3 ± 0.01 and 2.1 ± 0.1 μgC/g/h) to subsequently decrease to the basal level of respiration after 30 days (1 ± 0.01 and 0.9 ± 0.01 μgC/g/h in low and high F:B soil, respectively). ^13^C in respired CO_2_ in low and high F:B soil was 163.1 ± 2.9 and 133.9 ± 11.7 ng^13^C/g/h at day 1, 229.2 ± 2.8 and 144.6 ± 11 ng^13^C/g/h at day 7, and 26.8 ± 0.3 and 29.4 ± 1.4 ng^13^C/g/h, respectively, at day 30 (**Figure 4B**). ^13^C in respired CO_2_ was consistently higher in low F:B soil in comparison to high F:B soil; global effect of soil class (*p* = 0.02), time (*p* < 0.0001) and both (*p* = 0.01) was significant. Plant litter ^13^C that gets incorporated into SOM was estimated by subtracting the ^13^C excess in microbial biomass from that of bulk soil organic matter. ^13^C in non-living SOM was 2.3 ± 0.4 and 3 ± 0.5 μg^13^C/g at day 3 and 3.1 ± 0.6 and 5.9 ± 1.7 μg^13^C/g at day 30, in low and high F:B soil, respectively (**Figure 4C**). A mass balance of applied litter-derived ^13^C (531.3 ± 21.5 μg^13^C) into the different soil pools was performed at 30 days after litter addition. The total respiratory loss over the time period under investigation was estimated at 302 ± 6.2 and 244.8 ± 2.7 μg^13^C in low and high F:B soil, respectively. After 30 days, the ^13^C in microbial biomass was 10.7 ± 0.1 and 13.5 ± 0.4 μg^13^C; and that in non-living SOM was 151.1 ± 30.3 and 297 ± 85 μg^13^C in low and high F:B soil, respectively.

### Litter Input Driven Shifts in Microbial Transcriptome and Proteome

RNA-Seq derived functional information (38644 KEGG annotated functions) at different hierarchical classes was used to discern the total microbial response to litter addition. In general, the fold change in transcripts after litter input was very small (**Supplementary Figure S8**), hence, it was not possible to tear apart the differences in functional response of the low and high F:B soil microbial community. It is worthwhile to note here that the abundances of these transcripts at the lowest level of functional classification were quite low due to lack of sequencing depth. Database annotation of the extracted proteins resulted in 1318 identified protein groups. In comparison to the shift in transcript abundance, the fold change in proteins on litter application was larger (**Supplementary Figure S8**). The functional information derived from the omics techniques was not robust hence not presented and discussed in this report.

## Discussion

We hypothesized that soil microbial communities’ F:B ratio is linked to its capacity to store carbon; with higher F:B dominance being linked to higher carbon sequestration. To prove this hypothesis, we performed a ^13^C labeled litter degradation experiment on two soils that differed in their microbial community composition, specifically the F:B ratio, but were similar in their physical and chemical properties. While it is not entirely clear what caused the differences in the F:B ratio in the two soils, it appears to be due to subtle differences in the aboveground plant species diversity. It is likely that the higher plant species diversity caused higher F:B ratio of the microbial community due to ecological complementarity effects such as higher and diverse supply of resources for microorganisms (Lange et al., 2014). The effect of legumes and C:N ratio that are both intertwined could be ruled out because the number of plant functional groups were identical in the two soils. Soil texture, pH, moisture, organic C and N content and C:N ratio were largely similar in the two soils. Therefore these soils were ideal to investigate the standalone effect of differences in soil microbial communities’ F:B ratio on soil C storage.

The soils were chosen based on the F:B biomass ratio derived from PLFA analysis. Bacterial biomass was largely similar in the two communities, which makes the experimental system appropriate to investigate effects of fungal abundance and physiology. The soils were sieved and homogenized before setting up the mesocosm experiment that could have caused the fungal mycelia to break. They were left undisturbed for 3 weeks for the microbial community to acclimatize before plant litter was added to the mesocosms. F:B ratio was estimated again using RNA sequencing and protein profiling and both techniques confirm the initial finding; highlighting the congruence between lipid, RNA and protein biomarker approaches. Although the estimates of F:B biomass as well as abundance ratios are not identical they are largely in line with each other and thus any technical biases arising from the different methods are minimal (Strickland and Rousk, 2010).

RNA and protein based approaches were used to identify the microbial decomposers involved in litter degradation, both give information on active organisms and therefore it was possible to monitor time dependent shifts. F:B ratio derived from RNA-Seq suggests that F:B dominance increases on litter addition, more so in high F:B soil, clearly implicating fungal decomposers in substrate use; this was further substantiated by protein profiling. It is essential to note that while PLFA analysis-based fungal biomass (not with mesocosm soils but field samples) as a proportion of total microbial biomass was higher at 3–4%, relative fungal abundance based on RNA-Seq and proteomics was 1–3% and 0.5–2%, respectively. This highlights that fungi are underrepresented with nucleic acid and protein based approaches. This bias against fungi could be attributed to various reasons such as insufficient sequence database (Nilsson et al., 2006), poor extraction efficiencies of fungal proteins and nucleic acids (Feinstein et al., 2009), or fungal physiology of biomass under-allocation of these macromolecules.

The shift in relative abundance of different microbial phyla provides detailed information of the major decomposers of plant foliar litter. *Ascomycota* and *Basidiomycota* were identified as the major fungal decomposers with biggest fold change in abundance that is in line with the literature (Osono and Takeda, 2006; Osono, 2007; Schneider et al., 2012; Štursová et al., 2012). Among bacteria, *Acidobacteria* and *Actinobacteria* both appear to be important decomposers with increased abundance on litter application; which has been reported previously (Kirby, 2005; Baldrian et al., 2012). Interestingly, RNA-Seq derived *Actinobacteria*: rest of bacteria (A:RB) ratio increased only in low F:B soil suggesting that *Actinobacteria* take up the role of fungi in litter degradation when fungal abundance is lower. Such a shift has been previously observed in a fungicide treated soil where an increased proportion of tracer-derived carbon was quantified in *Actinobacteria* (Helfrich et al., 2015). A decrease in Actinobacterial proteins was observed in both soils, which was unexpected considering *Actinobacteria* are known to be important decomposers of complex organic matter (Kirby, 2005). On the contrary, Acidobacterial proteins and not transcripts increased in abundance on litter application. Acidobacteria have been previously linked to litter degradation (Schneider et al., 2012). Archaeabacterial abundance decreased on litter addition suggesting its minor role in decomposition of complex substrates, there is no proof that they play a major role in organic matter decomposition in soil (Offre et al., 2013). Overall, the shifts in microbial groups on litter application suggest that fungi played a significant role in plant litter degradation in this experiment.

To assess short term C storage, ^13^C labeled foliar litter was added to root-free soils and traced into respired CO_2_, microbial biomass and bulk soil organic matter (SOM). ^13^C enriched foliar litter added to root-free soils allows assessment of the fate of plant C in soils, in this case with differing F:B dominance. Litter derived ^13^C in respired CO_2_ was consistently lower in high F:B soil than low F:B soil, indicating that high F:B soils studied here loose less carbon through respiration in comparison to low F:B soils. Rate of respiratory loss was highest between 4 and 14 days and returned to basal level in 30 days after litter application. This suggests that most of the substrate (dried and powdered plant litter) was degraded within 30 days; similar decomposition dynamics have been reported by other authors (Thiessen et al., 2013). Microbial biomass increased on litter addition but stayed rather uniform throughout the experimental period. It is likely that the added litter carbon feeds the soil microbial loop that leads to cross-feeding and reuse of organic carbon (Gleixner, 2013; Apostel et al., 2015; Basler et al., 2015; Malik et al., 2015). Higher amounts of ^13^C litter in non-living SOM was observed in F:B dominated soil, which could arise from a greater microbial contribution to SOM formation or smaller net mass loss of added litter. In either case, it would mean higher short-term soil carbon storage in soils with F:B dominance. It is more likely that the higher incorporation of litter-derived carbon into SOM in high F:B soils is through microbial necromass contribution, because we observed an increase in the ^13^C excess in non-living SOM in the later stages of litter decomposition (Rubino et al., 2010; Cotrufo et al., 2015; Lange et al., 2015). However, we cannot demonstrate this transfer of ^13^C from plant litter into SOC through microbial biomass as CFE derived microbial biomass extraction is known to be incomplete and selective (Malik et al., 2016); and it is likely that we are missing a fraction of the microbial pool.

After a month of litter application, 65.1 ± 1.3% of litter ^13^C was lost by microbial respiration in low F:B soil, while only 44 ± 0.5% was lost in high F:B soil. The fraction in microbial biomass was largely similar at 2.3 ± 0.03% and 2.4 ± 0.08% in low and high F:B soil, respectively. To summarize, high F:B soil communities are possibly more efficient with substrate use over the experimental time period as the respiratory loss is lower. The iterative process of microbial growth, replication and death leads to incorporation of microbial cell fragments, residues and discharges into SOM (Miltner et al., 2012; Gleixner, 2013). The amount of litter derived ^13^C incorporated into SOM a month after litter addition was higher in high F:B soil at 53.5 ± 15.3% compared to 32.6 ± 6.5% in low F:B soil. These multitudes of evidences suggest that the F:B dominance in microbial communities can be linked to a higher soil C storage potential.

Despite bacteria being most dominant in the studied soil system, there was clear consistency in RNA and protein-based analyses confirming an increased fungal abundance on litter addition. We found evidence from the litter degradation experiment in the grassland soil under investigation to support the hypothesis that higher fungal dominance leads to higher soil C storage potential. A smaller respiratory loss in microbial communities with high F:B ratio was linked to higher amount of litter derived C transformation to SOC. Here we provide evidence to support the ‘community change’ hypothesis, which suggests that a shift in certain microbial functional groups are more likely to cause soil carbon accumulation due to their physiological capabilities. Microbial functional response specifically linked to the central carbon metabolism explored using transcriptomics and/or proteomics would be the next step to understand the physiological mechanisms of organic matter degradation in soil. A key hypothesis to test in future studies would be that certain microbial groups channel more carbon into biosynthetic pathways than into carbon transformations that lead to release of CO_2_. Such a balance of microbial anabolic and catabolic processes often referred to as microbial carbon use efficiency can be linked to the capacity of soil microorganisms to regulate the flow of C in soil systems (Dijkstra et al., 2011). A more efficient physiological pathway with higher anabolic fluxes could lead to increased microbial biomass and consequently higher soil carbon storage (Lange et al., 2015). Given the indication here that fungi may be important regulators of these processes, we recognize that new molecular advancements are required to specifically assess the functional genes derived from fungi, which are typically at very low abundance in soil microbial RNA extracts (Malik et al., 2015). We therefore strongly endorse such multi-disciplinary approaches and technological advancements to explore the relationship between active microbial communities and their function to gain a better mechanistic understanding of microbial-driven C cycling processes and to linking microbial identity to ecosystem functioning.

## Author Contributions

AM, RG, and GG designed research; AM and VS performed the experiment and stable isotope analyses; SC, AM, and NJ performed proteomic analysis; AM and AO performed RNA-Seq; PV performed PLFA analysis; AM and JP performed statistical analysis; AM and RG drafted the manuscript; RG, NJ, MvB, and GG contributed new reagents and analytical tools; all authors were involved in critical revision and approval of the final version.

## Conflict of Interest Statement

The authors declare that the research was conducted in the absence of any commercial or financial relationships that could be construed as a potential conflict of interest.

## Footnotes

1 http://www.prophane.de/.

## References

[B1] Allison S. D. Martiny J. B. H. (2008). Resistance, resilience, and redundancy in microbial communities. *Proc. Natl. Acad. Sci. U. S. A.* 105 11512–11519. 10.1073/pnas.0801925105 18695234 PMC2556421

[B2] Amundson R. (2001). The carbon budget in soils. *Annu. Rev. Earth Planet. Sci.* 29 535–562. 10.1146/annurev.earth.29.1.535

[B3] Apostel C. Dippold M. Kuzyakov Y. (2015). Biochemistry of hexose and pentose transformations in soil analyzed by position-specific labeling and 13C-PLFA. *Soil Biol. Biochem.* 80 199–208. 10.1016/j.soilbio.2014.09.005

[B4] Bailey V. L. Smith J. L. Bolton H. (2002). Fungal-to-bacterial ratios in soils investigated for enhanced C sequestration. *Soil Biol. Biochem.* 34 997–1007. 10.1016/S0038-0717(02)00033-0

[B5] Baldrian P. Kolařík M. Štursová M. Kopecký J. Valášková V. Větrovský T. (2012). Active and total microbial communities in forest soil are largely different and highly stratified during decomposition. *ISME J.* 6 248–258. 10.1038/ismej.2011.95 21776033 PMC3260513

[B6] Bardgett R. D. Freeman C. Ostle N. J. (2008). Microbial contributions to climate change through carbon cycle feedbacks. *ISME J.* 2 805–814. 10.1038/ismej.2008.58 18615117

[B7] Bardgett R. D. Hobbs P. J. Frostegård Å. (1996). Changes in soil fungal:bacterial biomass ratios following reductions in the intensity of management of an upland grassland. *Biol. Fertil. Soils* 22 261–264. 10.1007/s003740050108

[B8] Basler A. Dippold M. Helfrich M. Dyckmans J. (2015). Microbial carbon recycling: an underestimated process controlling soil carbon dynamics. *Biogeosci. Discuss* 12 9729–9750. 10.5194/bgd-12-9729-2015

[B9] Billings S. A. Ballantyne F. (2013). How interactions between microbial resource demands, soil organic matter stoichiometry, and substrate reactivity determine the direction and magnitude of soil respiratory responses to warming. *Glob. Chang. Biol.* 19 90–102. 10.1111/gcb.12029 23504723

[B10] Bligh E. G. Dyer W. J. (1959). A rapid method of total lipid extraction and purification. *Can. J. Biochem. Physiol.* 37 911–917. 10.1139/o59-099 13671378

[B11] Boschker H. T. S. Nold S. C. Wellsbury P. Bos D. de Graaf W. Pel R. (1998). Direct linking of microbial populations to specific biogeochemical processes by 13C-labelling of biomarkers. *Nature* 392 396–400. 10.1038/33900

[B12] Cotrufo M. F. Soong J. L. Horton A. J. Campbell E. E. Haddix M. L. Wall D. H. (2015). Formation of soil organic matter via biochemical and physical pathways of litter mass loss. *Nat. Geosci.* 8 776–779. 10.1038/ngeo2520

[B13] Davidson E. A. Janssens I. A. (2006). Temperature sensitivity of soil carbon decomposition and feedbacks to climate change. *Nature* 440 165–173. 10.1038/nature04514 16525463

[B14] Dijkstra P. Blankinship J. C. Selmants P. C. Hart S. C. Koch G. W. Schwartz E. (2011). Probing carbon flux patterns through soil microbial metabolic networks using parallel position-specific tracer labeling. *Soil Biol. Biochem.* 43 126–132. 10.1016/j.soilbio.2010.09.022

[B15] Feinstein L. M. Sul W. J. Blackwood C. B. (2009). Assessment of bias associated with incomplete extraction of microbial DNA from soil. *Appl. Environ. Microbiol.* 75 5428–33. 10.1128/AEM.00120-09 19561189 PMC2725469

[B16] Gleixner G. (2013). Soil organic matter dynamics: a biological perspective derived from the use of compound-specific isotopes studies. *Ecol. Res.* 28 683–695. 10.1007/s11284-012-1022-9

[B17] Helfrich M. Ludwig B. Thoms C. Gleixner G. Flessa H. (2015). The role of soil fungi and bacteria in plant litter decomposition and macroaggregate formation determined using phospholipid fatty acids. *Appl. Soil Ecol.* 96 261–264. 10.1016/j.apsoil.2015.08.023

[B18] Kirby R. (2005). Actinomycetes and lignin degradation. *Adv. Appl. Microbiol.* 58 125–168. 10.1016/S0065-2164(05)58004-3 16543032

[B19] Kramer C. Gleixner G. (2008). Soil organic matter in soil depth profiles: distinct carbon preferences of microbial groups during carbon transformation. *Soil Biol. Biochem.* 40 425–433. 10.1016/j.soilbio.2007.09.016

[B20] Lal R. (2010). Managing soils for a warming earth in a food-insecure and energy-starved world. *J. Plant Nutr. Soil Sci.* 173 4–15. 10.1002/jpln.200900290

[B21] Lange M. Eisenhauer N. Sierra C. A. Bessler H. Engels C. Griffiths R. I. (2015). Plant diversity increases soil microbial activity and soil carbon storage. *Nat. Commun.* 6:6707 10.1038/ncomms7707 25848862

[B22] Lange M. Habekost M. Eisenhauer N. Roscher C. Bessler H. Engels C. (2014). Biotic and abiotic properties mediating plant diversity effects on soil microbial communities in an experimental grassland. *PLoS ONE* 9:e96182 10.1371/journal.pone.0096182 PMC4015938 24816860

[B23] Legendre P. Gallagher E. D. (2001). Ecologically meaningful transformations for ordination of species data. *Oecologia* 129 271–280. 10.1007/s004420100716 28547606

[B24] Malik A. Blagodatskaya E. Gleixner G. (2013). Soil microbial carbon turnover decreases with increasing molecular size. *Soil Biol. Biochem.* 62 115–118. 10.1016/j.soilbio.2013.02.022

[B25] Malik A. A. Dannert H. Griffiths R. I. Thomson B. C. Gleixner G. (2015). Rhizosphere bacterial carbon turnover is higher in nucleic acids than membrane lipids: implications for understanding soil carbon cycling. *Front. Microbiol.* 6:268 10.3389/fmicb.2015.00268 PMC4391234 25914679

[B26] Malik A. A. Roth V.-N. Hébert M. Tremblay L. Dittmar T. Gleixner G. (2016). Linking molecular size, composition and carbon turnover of extractable soil microbial compounds. *Soil Biol. Biochem.* 100 66–73. 10.1016/j.soilbio.2016.05.019

[B27] Miltner A. Bombach P. Schmidt-Brücken B. Kästner M. (2012). SOM genesis: microbial biomass as a significant source. *Biogeochemistry* 111 41–55. 10.1007/s10533-011-9658-z

[B28] Nilsson R. H. Ryberg M. Kristiansson E. Abarenkov K. Larsson K. -H. Kõljalg U. (2006). Taxonomic reliability of DNA sequences in public sequence databases: a fungal perspective. *PLoS ONE* 1:e59 10.1371/journal.pone.0000059 PMC1762357 17183689

[B29] Offre P. Spang A. Schleper C. (2013). Archaea in biogeochemical cycles. *Annu. Rev. Microbiol.* 67 437–457. 10.1146/annurev-micro-092412-155614 23808334

[B30] Osono T. (2007). Ecology of ligninolytic fungi associated with leaf litter decomposition. *Ecol. Res.* 22 955–974. 10.1007/s11284-007-0390-z

[B31] Osono T. Takeda H. (2006). Fungal decomposition of *Abies* needle and *Betula* leaf litter. *Mycologia* 98 172–179. 10.3852/mycologia.98.2.172 16894962

[B32] Parks D. H. Tyson G. W. Hugenholtz P. Beiko R. G. (2014). STAMP: statistical analysis of taxonomic and functional profiles. *Bioinformatics* 30 3123–3124. 10.1093/bioinformatics/btu494 25061070 PMC4609014

[B33] Pinheiro J. Bates D. DebRoy S. Sarkar D. R Core Team (2014). *nlme: Linear and Nonlinear Mixed Effects Models. R Package Version 3.* 1–117. Available at: http://cran.r-project.org/web/packages/nlme/index.html

[B34] R Development Core Team (2013). *R: A Language and Environment for Statistical Computing.* Vienna: The R Foundation for Statistical Computing Available at: http://www.R-project.org/

[B35] Roscher C. Schumacher J. Baade J. Wilcke W. Gleixner G. Weisser W. W. (2004). The role of biodiversity for element cycling and trophic interactions: an experimental approach in a grassland community. *Basic Appl. Ecol.* 5 107–121. 10.1078/1439-1791-00216

[B36] Rubino M. Dungait J. A. J. Evershed R. P. Bertolini T. De Angelis P. D’Onofrio A. (2010). Carbon input belowground is the major C flux contributing to leaf litter mass loss: evidences from a 13C labelled-leaf litter experiment. *Soil Biol. Biochem.* 42 1009–1016. 10.1016/j.soilbio.2010.02.018

[B37] Schimel J. (2013). Soil carbon: microbes and global carbon. *Nat. Clim. Chang.* 3 867–868. 10.1038/nclimate2015

[B38] Schimel J. P. Schaeffer S. M. (2012). Microbial control over carbon cycling in soil. *Front. Microbiol.* 3:348 10.3389/fmicb.2012.00348 PMC3458434 23055998

[B39] Schneider T. Keiblinger K. M. Schmid E. Sterflinger-Gleixner K. Ellersdorfer G. Roschitzki B. (2012). Who is who in litter decomposition? Metaproteomics reveals major microbial players and their biogeochemical functions. *ISME J.* 6 1749–1762. 10.1038/ismej.2012.11 22402400 PMC3498922

[B40] Sinsabaugh R. L. Manzoni S. Moorhead D. L. Richter A. (2013). Carbon use efficiency of microbial communities: stoichiometry, methodology and modelling. *Ecol. Lett.* 16 930–939. 10.1111/ele.12113 23627730

[B41] Six J. Frey S. D. Thiet R. K. Batten K. M. (2006). Bacterial and fungal contributions to carbon sequestration in agroecosystems. *Soil Sci. Soc. Am. J.* 70 555–569. 10.2136/sssaj2004.0347

[B42] Strickland M. S. Rousk J. (2010). Considering fungal:bacterial dominance in soils – Methods, controls, and ecosystem implications. *Soil Biol. Biochem.* 42 1385–1395. 10.1016/j.soilbio.2010.05.007

[B43] Štursová M. Žifčáková L. Leigh M. B. Burgess R. Baldrian P. (2012). Cellulose utilization in forest litter and soil: identification of bacterial and fungal decomposers. *FEMS Microbiol. Ecol.* 80 735–746. 10.1111/j.1574-6941.2012.01343.x 22379979

[B44] Thiessen S. Gleixner G. Wutzler T. Reichstein M. (2013). Both priming and temperature sensitivity of soil organic matter decomposition depend on microbial biomass – An incubation study. *Soil Biol. Biochem.* 57 739–748. 10.1016/j.soilbio.2012.10.029

[B45] Urich T. Lanzén A. Qi J. Huson D. H. Schleper C. Schuster S. C. (2008). Simultaneous assessment of soil microbial community structure and function through analysis of the meta-transcriptome. *PLoS ONE* 3:e2527 10.1371/journal.pone.0002527 PMC2424134 18575584

[B46] Vance E. D. Brookes P. C. Jenkinson D. S. (1987). An extraction method for measuring soil microbial biomass C. *Soil Biol. Biochem.* 19 703–707. 10.1016/0038-0717(87)90052-6

[B47] Waring B. G. Averill C. Hawkes C. V. (2013). Differences in fungal and bacterial physiology alter soil carbon and nitrogen cycling: insights from meta-analysis and theoretical models. *Ecol. Lett.* 16 887–894. 10.1111/ele.12125 23692657

[B48] Wieder W. R. Bonan G. B. Allison S. D. (2013). Global soil carbon projections are improved by modelling microbial processes. *Nat. Clim. Chang.* 3 909–912. 10.1038/nclimate1951

[B49] Zhou J. Xue K. Xie J. Deng Y. Wu L. Cheng X. (2011). Microbial mediation of carbon-cycle feedbacks to climate warming. *Nat. Clim. Chang.* 2 106–110. 10.1038/nclimate1331

