# Supplementary material for: Soil Fungal:Bacterial Ratios Are Linked to Altered Carbon Cycling
Source: Front Microbiol. 2016 Aug 9;7:1247. doi: 10.3389/fmicb.2016.01247 (PMC4977315; doi:10.3389/fmicb.2016.01247)

Supplementary Material

Soil fungal:bacterial ratios are linked to altered carbon cycling

Ashish A. Malik*, Somak Chowdhury, Veronika Schlager, Anna Oliver, Jeremy Puissant, Perla G. Mellado Vazquez, Nico Jehmlich, Martin von Bergen, Robert I. Griffiths, Gerd Gleixner

*** Correspondence:** ashmalik@ceh.ac.uk

Supplementary Figures

Figure S-1: Organic C, N and C:N ratio of the soils (0-10 cm depth) used in the experiment.

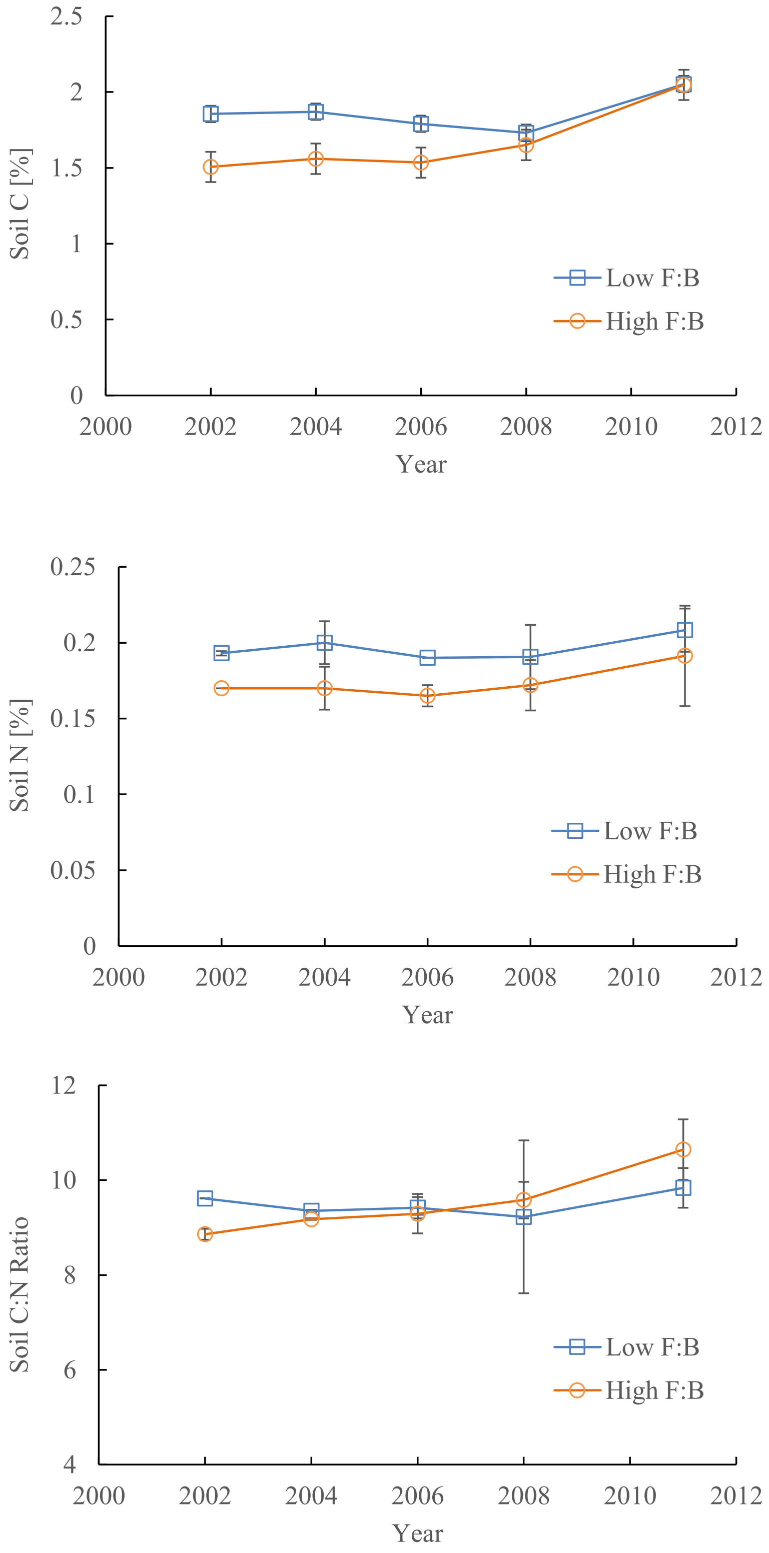

Figure S-2: Relative abundance of the major phyla in the two soils and the shifts on litter addition obtained using RNA-Seq based taxonomic classification

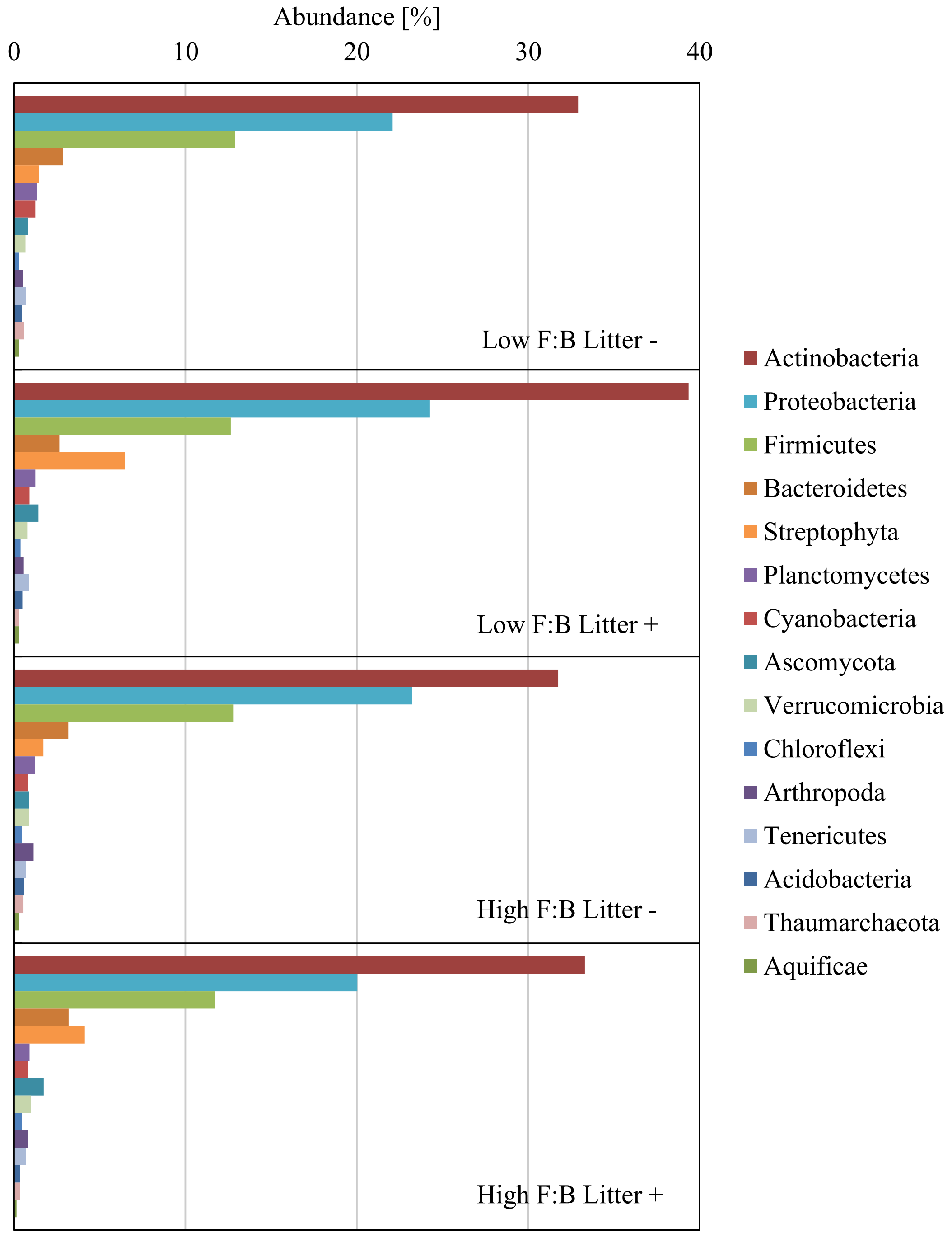

Figure S-3: RNA-Seq derived relative abundance of bacterial and eukaryotic domains in the two soils and their shift on litter addition. Global effect of F:B ratio and litter addition was assessed by repeated measures ANOVA.

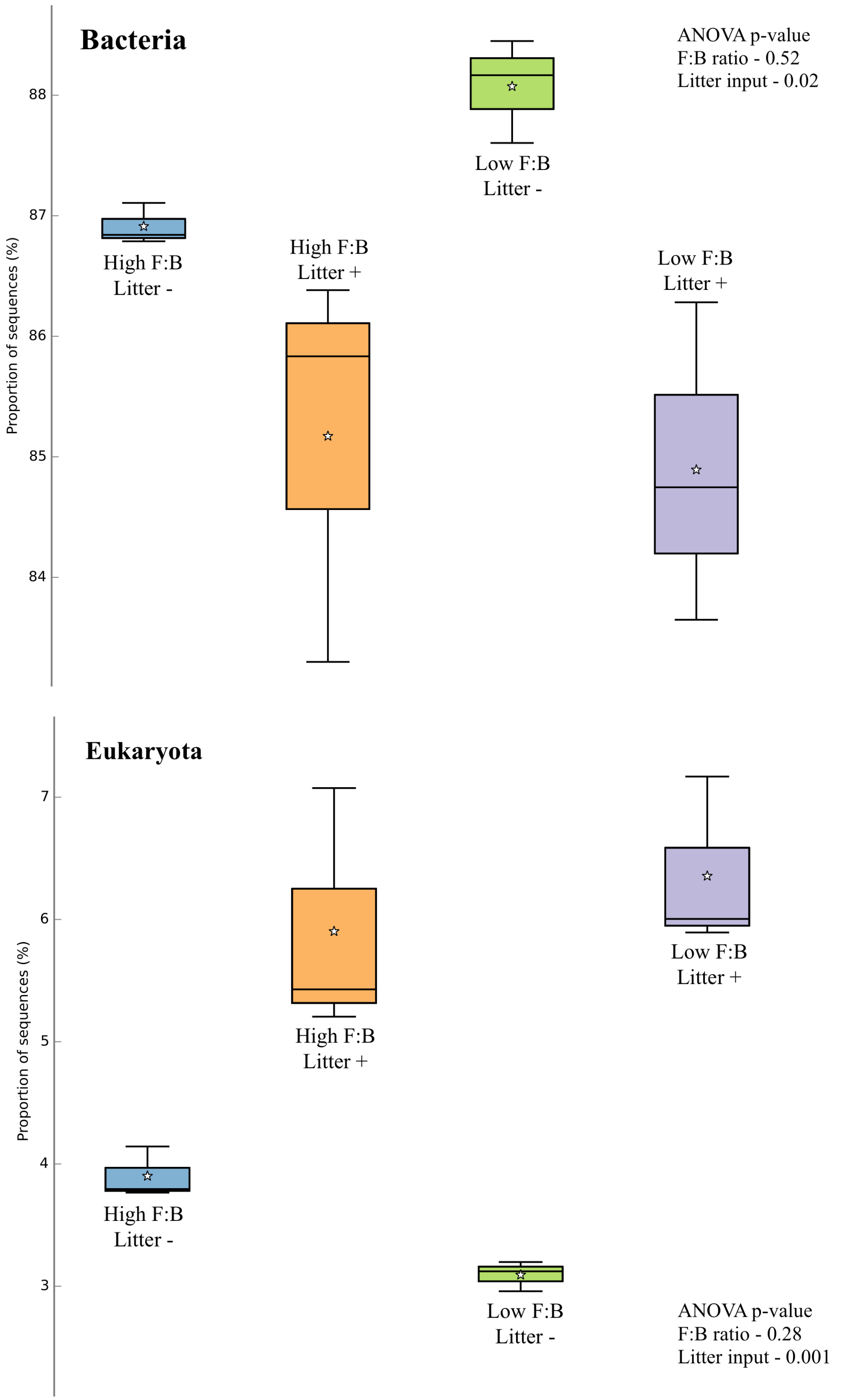

Figure S-4: RNA-Seq derived relative abundance of major fungal phyla in the two soils and their shift on litter addition. Global effect of F:B ratio and litter addition was assessed by repeated measures ANOVA.

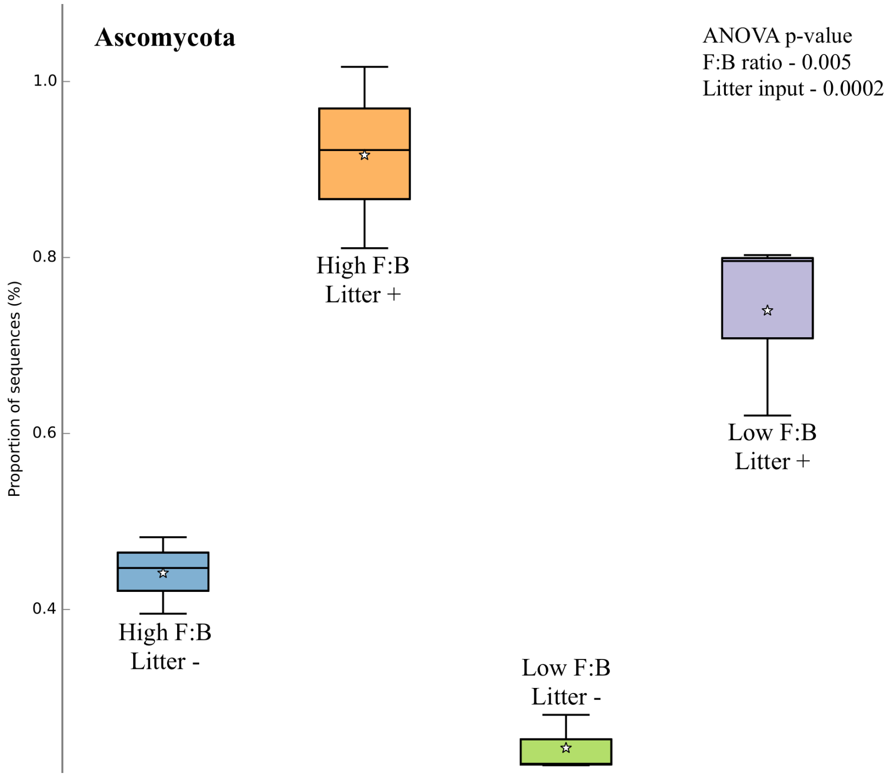

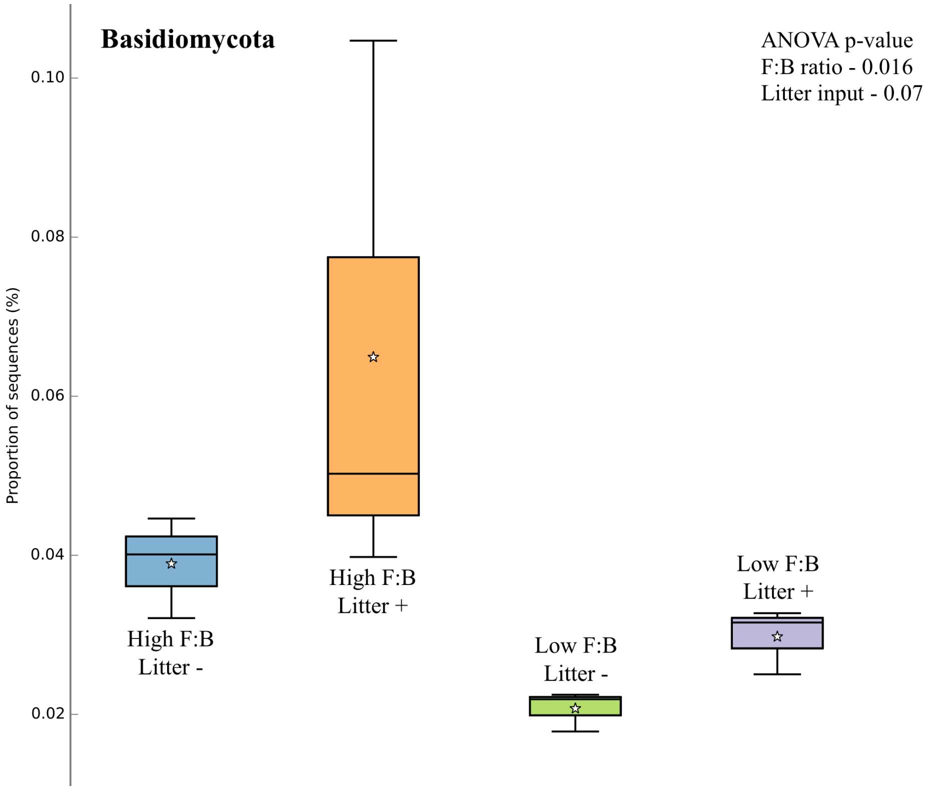

Figure S-5: Actinobacteria: rest of bacteria (A:RB) ratio estimated using RNA-Seq. Error bars represent standard error (n=3), lowercase letters from Fischer LSD test; treatments sharing a letter are not significant.

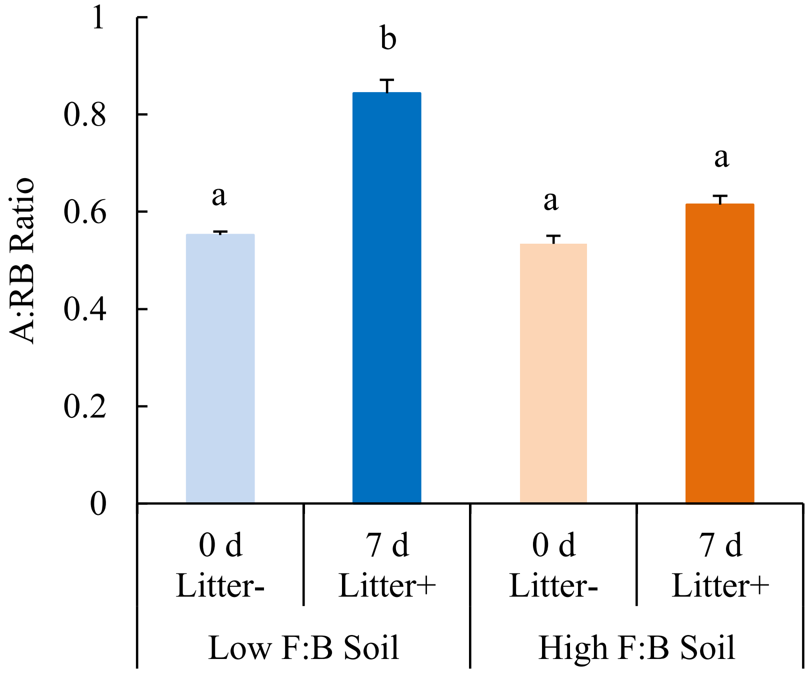

Figure S-6: Temporal trend in the rate microbial respiration. Note: error bars represent standard error; n=3.

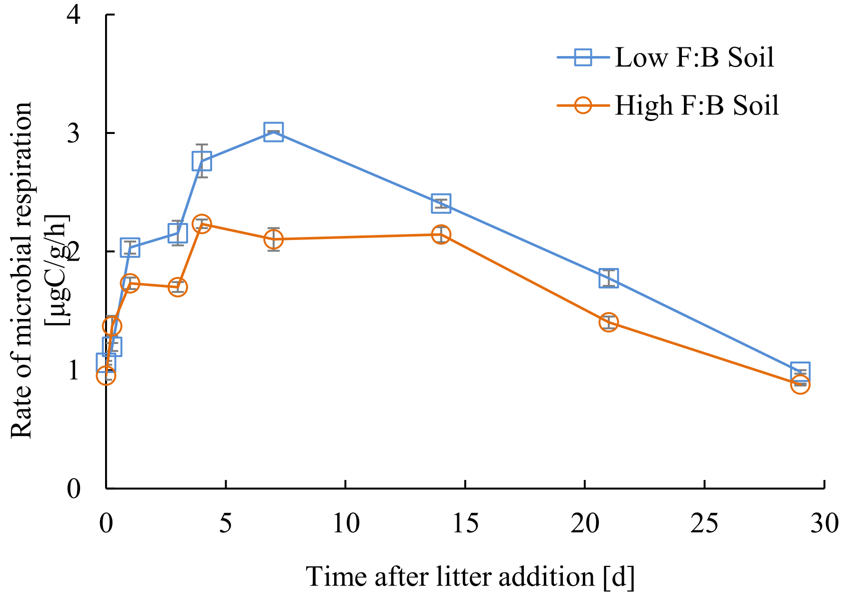

Figure S-7: δ^{13}C excess in microbial respired CO_2 and its temporal trend over the first 24h after litter addition. Note: error bars represent standard error; n=3.

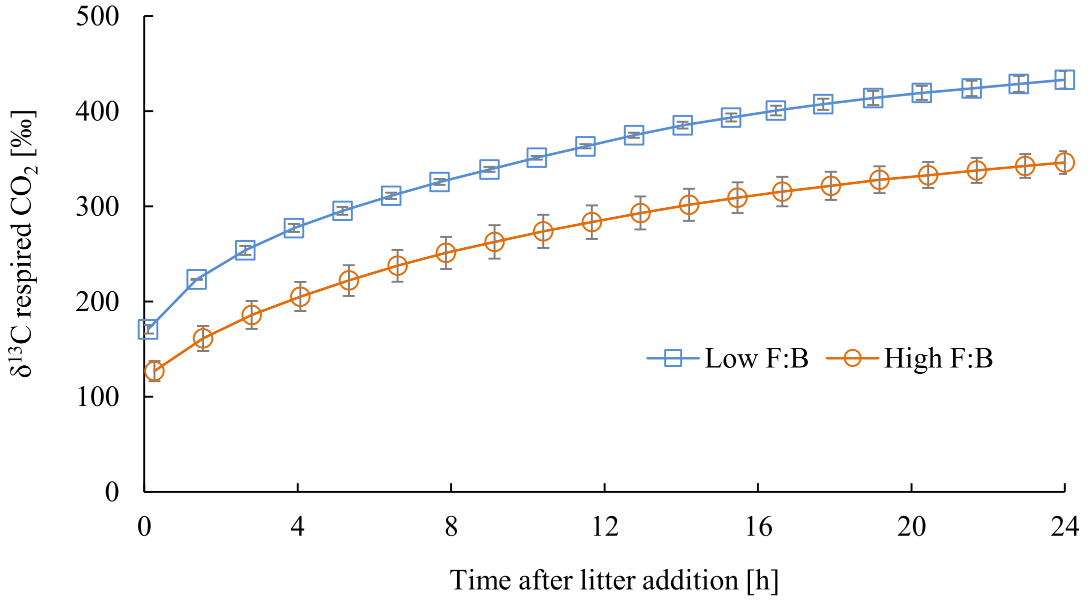

Figure S-8: Shifts in abundance of different COG/KOG functional groups after litter addition at different time points obtained using: (A) RNA-Seq: error bars represent standard error (n=3), and (B) Proteomics, values were derived from composite samples of replicates at each time point.

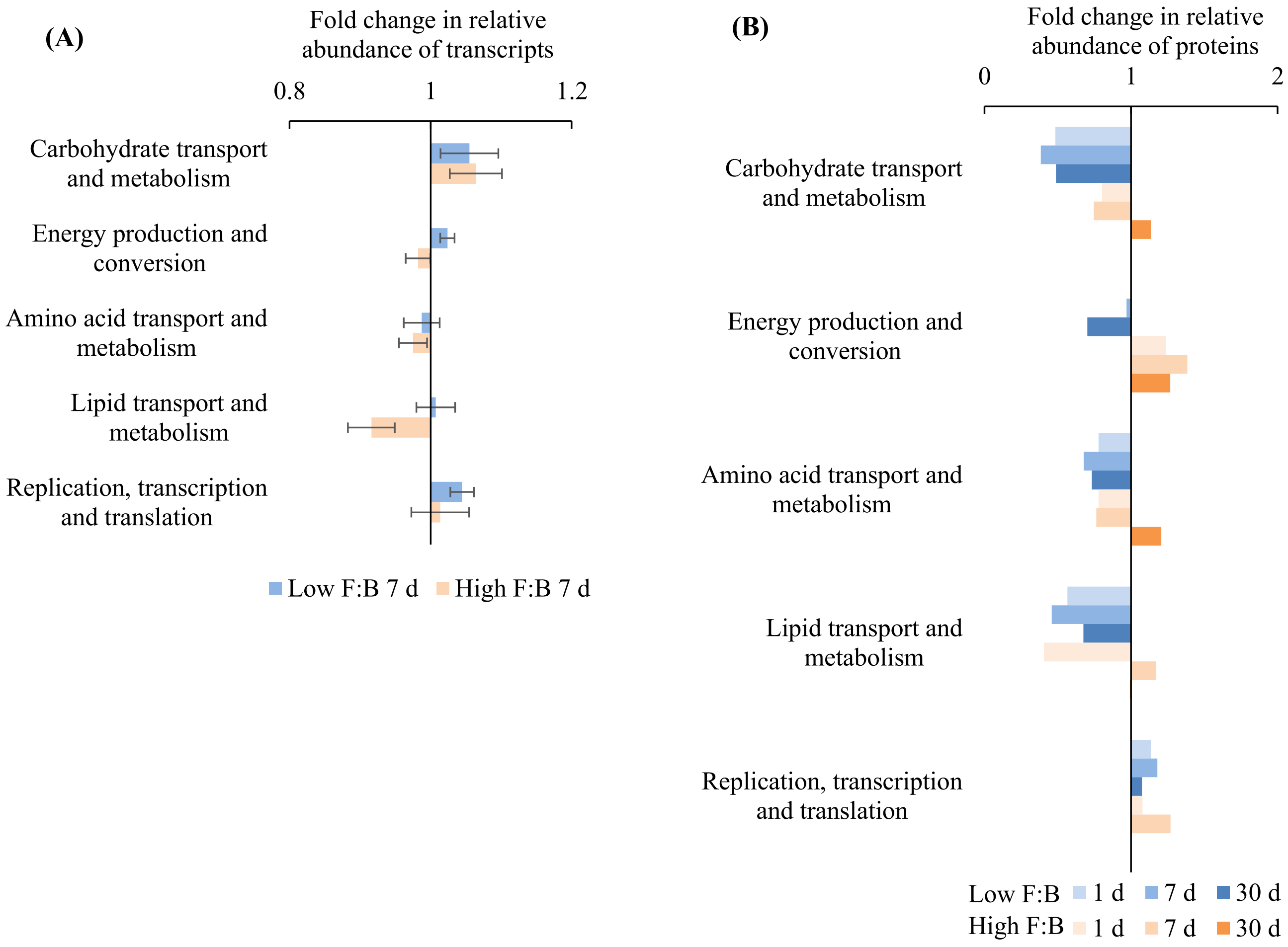

Supplement: Supplementary file 1 [file Image_1.PDF]
